# The blood monocyte to high density lipoprotein cholesterol ratio (MHR) is a possible marker of carotid artery plaque

**DOI:** 10.1186/s12944-022-01741-8

**Published:** 2022-12-03

**Authors:** Jie Xi, Shasha Men, Jingzhu Nan, Qiuliang Yang, Jin Dong

**Affiliations:** 1 Center of Translational Medicine Research, Medical Innovation Research Department of Chinese PLA General Hospital, Beijing, 100853 China; 2 Department of Clinical Laboratory, the 1st Medical Centre, Chinese PLA General Hospital, Beijing, 100853 China

**Keywords:** MHR, Carotid plaque, Severity, Atherosclerosis, Inflammation, Metabolic indexes

## Abstract

**Background:**

MHR is the ratio of monocyte to high-density lipoprotein cholesterol (HDL-C). It has been reported that MHR changes are associated with cardiovascular and cerebrovascular disease. Carotid plaque is a common vascular lesion of the carotid artery and is a manifestation of atherogenesis. This study investigated the relationships between the MHR and the incidence of carotid plaques.

**Methods:**

The data of 3848 physical examiners were analyzed for retrospective analysis, which included 1428 patients with noncarotid plaque, 1133 patients with single carotid plaque, and 1287 patients with bilateral or multiple carotid plaques. Statistical analysis was performed on SPSS 22.0 0 software and statistical software R and its GAM package.

**Results:**

The difference was statistically significant in the levels of MHR, body mass index (BMI), high-sensitivity C-reactive protein (hs-CRP), blood lipids (HDL-C, low-density lipoprotein cholesterol (LDL-C), total cholesterol (TC), triglyceride (Tg)), blood glucose (Glu), hemoglobin A1c (HbA1c), renal function (urea, creatinine (Crea)), estimated glomerular filtration rate (eGFR), and uric acid (Ua) in the carotid plaque groups (*P* < 0.001, respectively). There was no significant difference between the sex (*P* = 0.635) and age (*P* = 0.063) in the different groups. MHR levels were positively correlated with BMI (*r* = 0.364, *P* < 0.001), hs-CRP (*r* = 0.320, *P* < 0.001), Tg (*r* = 0.417, *P* < 0.001), Crea (*r* = 0.323, *P* < 0.001), eGFR (*r* = − 0.248, *P* < 0.001), Ua (*r* = 0.383, *P* < 0.001) and HbA1c (*r* = 0.197, *P* < 0.001). Levels of TC, Glu, and urea were slightly correlated with the MHR level (*r* = − 0.150, *P* < 0.001; *r* = 0.187, *P* < 0.001; *r* = 0.137, *P* < 0.001, respectively). The MHR level increased with elevated severity of carotid plaque in subjects without hypertension or diabetes (*P* < 0.001). In adjusted models, with the rise of MHR level, the probability of occurrence of carotid plaque had a 1.871-fold (95% CI: 1.015–3.450, *P* = 0.045) increase; the probability of multiple occurrences of carotid plaques had a 2.896-fold (95% CI: 1.415–5.928, *P* < 0.001) increase. The GAM curve showed a nonlinear correlation between the normalized MHR and the probability of carotid plaque occurrence.

**Conclusions:**

MHR could be used as a possible marker for plaque formation and severity.

## Introduction

Carotid plaque is a manifestation of carotid atherosclerosis and is related to atherosclerotic cardiovascular disease [1], increasing the risks of major adverse cardiac events (MACEs) [2, 3]. The abnormal metabolism of glucose and lipids is a source of inflammation, and the development of atherosclerosis is an inflammatory process [4].

Monocytes play a key role in atherosclerosis-related inflammatory responses and participate in all stages of atherosclerosis [5]. Studies have shown that monocytes are related to carotid intima-media thickness (IMT), carotid stenosis and neovascularization of carotid plaque [6, 7]. High-density lipoprotein cholesterol (HDL-C) can promote the reverse transport of cholesterol and has anti-inflammatory, antioxidant, antithrombotic and anti-atherosclerosis effects. Previous studies have shown that an increase in HDL-C levels is considered to be an important protective factor against cardiovascular disease [8]. However, recent studies have shown that the effect of HDL-C on cardiovascular disease is more complicated; elevated levels of HDL-C do not always have a protective effect [9], and HDL still plays a positive role in cardiovascular events. Studies have shown that the anti-inflammatory capacity of HDL reflects vascular protection against key steps in atherogenesis [10], and low levels of HDL increase the risk of cardiovascular disease [11]. Previous studies have found that monocytes and HDL-C may have a significant negative correlation in regulating atherosclerotic lesions [12]. Therefore, the monocyte to HDL-C ratio (MHR) can be used as a novel inflammatory marker to indicate the atherosclerotic inflammatory response and oxidative stress process in vivo [12, 13]. MHR correlates with the degree and distribution of intracranial and extracranial atherosclerotic stenosis [7]. Studies have shown that the MHR is a potential marker of cardiovascular disease [14, 15] and may be a valuable marker for assessing atherosclerotic-related diseases [12, 16, 17].

In China, carotid plaque examination is commonly performed with ultrasound Doppler technology in routine physical examination, and the existence of carotid plaque is reported as none, single site or multiple sites. The occurrence of carotid plaque is considered to be related to metabolic abnormalities and increases the risks of cardiovascular and cerebrovascular diseases. This study investigated the relationships between the MHR level and the incidence of carotid artery plaque and related blood parameter changes.

## Methods

### Study subjects, inclusion and exclusion criteria and group division

Data from physical examination personnel in the first medical center of the General Hospital of PLA from January to April 2018 were collected, and 3848 subjects were included for retrospective analysis. The exclusion criteria were as follows: (1) lack of imaging data; (2) imaging data or disease history showing the existence of cerebrovascular diseases, including aneurysm rupture, cavernous hemangioma, posttraumatic hemorrhage and hemorrhagic infarction histories; (3) incomplete blood parameters (blood routine examination and chemistry results); and (4) serious liver and kidney diseases, cardiovascular diseases, blood diseases or malignant tumors in this examination.

Based on the ultrasonic Doppler examination of carotid plaque, relevant laboratory data were collected according to the occurrence of carotid plaque and the site number of carotid plaque (severity). The study was a retrospective analysis of clinical data, and exemption from informed consent was approved by the ethics committee of Chinese PLA General Hospital. All experimental protocols were approved by the ethics committee of Chinese PLA General Hospital, and all methods were carried out in accordance with relevant guidelines and regulations.

The groups were divided based on the ultrasonic Doppler examination results, which included 1428 noncarotid plaque patients (control group), 1133 patients with single carotid plaque (SC group), and 1287 patients with bilateral or multiple carotid plaques (bilateral or multiple, MC group). Subjects with or without carotid plaque were also grouped for difference comparison.

### Clinical data and laboratory tests

The clinical data were collected, including age, sex, disease history, body mass index (BMI), monocyte level (M), white blood cell (WBC), high-sensitivity C-reactive protein (hs-CRP), blood glucose (Glu), hemoglobin A1c (HbA1c), high-density lipoprotein cholesterol (HDL-C), low-density lipoprotein cholesterol (LDL-C), total cholesterol (TC), triglyceride (Tg), urea, creatinine (Crea), estimated glomerular filtration rate (eGFR) and uric acid (Ua). The absolute monocyte count was used to calculate the MHR, and the MHR value was then calculated (MHR = M/HDL-C).

### Statistical analyses

Statistical analysis was performed on SPSS 22.0 0 software and statistical software R (https://www.r-project.org/, the R Foundation) and its GAM package. Continuous parameters of the data were tested with normal distribution. A nonnormal distribution was found, continuous parameters are shown as the median (25th percentile.75th percentile) in each group, and categorical parameters are shown as numbers and percentages. Continuous parameters in different groups were compared with nonparametric statistical methods (Mann-Whitney test for comparison of two groups and Kruskal-Wallis H test for comparison of three groups); category parameters were tested by Chi-square test. Spearman correlation tests were used between MHR levels and other parameters. Odds ratios (ORs, exp.(B)) and their 95% confidence intervals (CIs) for the independent association between MHR and the occurrence of carotid artery plaque were analyzed by binary logistic regression. The association between the MHR and the severity of carotid artery plaques was analyzed with multiple regression. A generalized additive model (GAM) was used for estimating nonlinear relationships of MHR values and occurrence of carotid artery plaque, and MHR values were transformed by rank of Normal Score using Blom’s Formula due to highly skewed distributions before GAM analyses. A *P* value under 0.05 was considered statistically significant.

## Results

### Comparison of MHR and other parameters in the control and carotid plaque groups

The difference was statistically significant in the levels of MHR, BMI, hs-CRP, blood lipids (HDL-C, LDL-C, TC, Tg), glucose, HbA1c, renal function (Urea, Crea, eGFR), and uric acid (Ua) in the control and carotid plaque groups (*P* < 0.001, respectively). There was no significant difference between sex (*P* = 0.635) and age (*P* = 0.063) in the different groups (shown in Table 1).

**Table 1:** Characteristics of subjects in different carotid plaque groups

Parameters	Control group	SC group	MC group	*P* value
Gender (n, total/female)	1428 (520)	1133 (392)	1287 (459)	0.635
Age, year	50 (44.55)	51 (46.56)	51 (45.55)	0.063
Disease history				
HT, n (%)	204 (14.28)	200 (17.65)	312 (24.24)	< 0.001
DM, n (%)	93 (6.51)	120 (10.59)	253 (19.65)	< 0.001
HT&DM, n (%)	23 (1.61)	30 (2.64)	71 (5.52)	< 0.001
SBP (mmHg)	121 (110.131)	125 (115.135)	130 (119.141)	< 0.001
DBP (mmHg)	80 (72.89)	84 (77.92)	86 (79.94)	< 0.001
Wight (kg)	68.6 (59.3.78.1)	72.5 (63.9.79.8)	73.7 (66.1.81.5)	< 0.001
Hight (cm)	167.6 (161.5.173.7)	169.5 (163.6.174.3)	170.1 (164.0.174.4)	< 0.001
BMI (kg/m^2^)	24.30 (22.30.26.60)	25.10 (23.30.27.20)	25.6 (23.80.27.80)	< 0.001
MHR, 10^9^/mmol/L	0.24 (0.17.0.34)	0.26 (0.19.0.36)	0.28 (0.21.0.38)	< 0.001
WBC, 10^9^/L	5.75 (4.88.6.78)	5.73 (4.88.6.74)	5.97 (5.07.7.14)	< 0.001
M, 10^9^/L	0.30 (0.24.0.38)	0.31 (0.25.0.39)	0.33 (0.27.0.42)	< 0.001
hs-CRP,mg/dL	0.09 (0.05.0.17)	0.10 (0.06.0.18)	0.11 (0.07.0.22)	< 0.001
Glu, mmol/L	5.35 (5.05.5.80)	5.51 (5.17.6.02)	5.71 (5.29.6.65)	< 0.001
HbA1c (%)	5.60 (5.40.5.90)	5.70 (5.50.6.10)	5.90 (5.60.6.50)	< 0.001
HDL-C, mmol/L	1.28 (1.06.1.53)	1.20 (1.02.1.44)	1.19 (1.01.1.39)	< 0.001
LDL-C, mmol/L	2.88 (2.38.3.35)	2.99 (2.49.3.53)	3.03 (2.50.3.58)	< 0.001
Tg, mmol/L	1.23 (0.88.1.86)	1.42 (1.05.2.07)	1.41 (1.03.2.03)	< 0.001
TC, mmol/L	4.61 (4.07.5.20)	4.77 (4.19.5.38)	4.75 (4.14.5.39)	< 0.001
Urea, mmol/L	4.82 (4.12.5.61)	5.00 (4.21.5.83)	5.19 (4.44.6.13)	< 0.001
Crea, umol/L	67.40 (56.43.78.08)	70.70 (60.25.79.90)	70.80 (62.40.80.30)	< 0.001
Ua, umol/L	320.10 (255.10.388.88)	340.90 (275.25.395.50)	339.20 (281.60.397.90)	< 0.001
eGFR,ml/(min·1.73m^2^)	107.33 (95.84.117.90)	106.27 (91.92.114.54)	104.4 (92.03.114.65)	< 0.001

SC group: subjects with single carotid plaque, MC group: subjects with bilateral or multiple carotid plaque. The difference was statistically significant at *P* < 0.05

*HT* hypertension, *DM* diabetes, *BMI* body mass index, *MHR* monocyte to HDL-C ratio, *WBC* white blood cell, *M* monocyte (absolute monocyte count), *hs-CRP* high-sensitivity C-reactive protein, *Glu* glucose, *HbA1c* hemoglobin A1c, *HDL-C* high-density lipoprotein cholesterol, *LDL-C* low-density lipoprotein cholesterol, *TC* total cholesterol, *Tg* triglyceride, *Crea* creatinine, *Ua* uric acid, *eGFR* estimated glomerular filtration rate. M = WBC * monocyte percentage

### Correlation analysis between MHR and other metabolic indexes

**Fig. 1:**
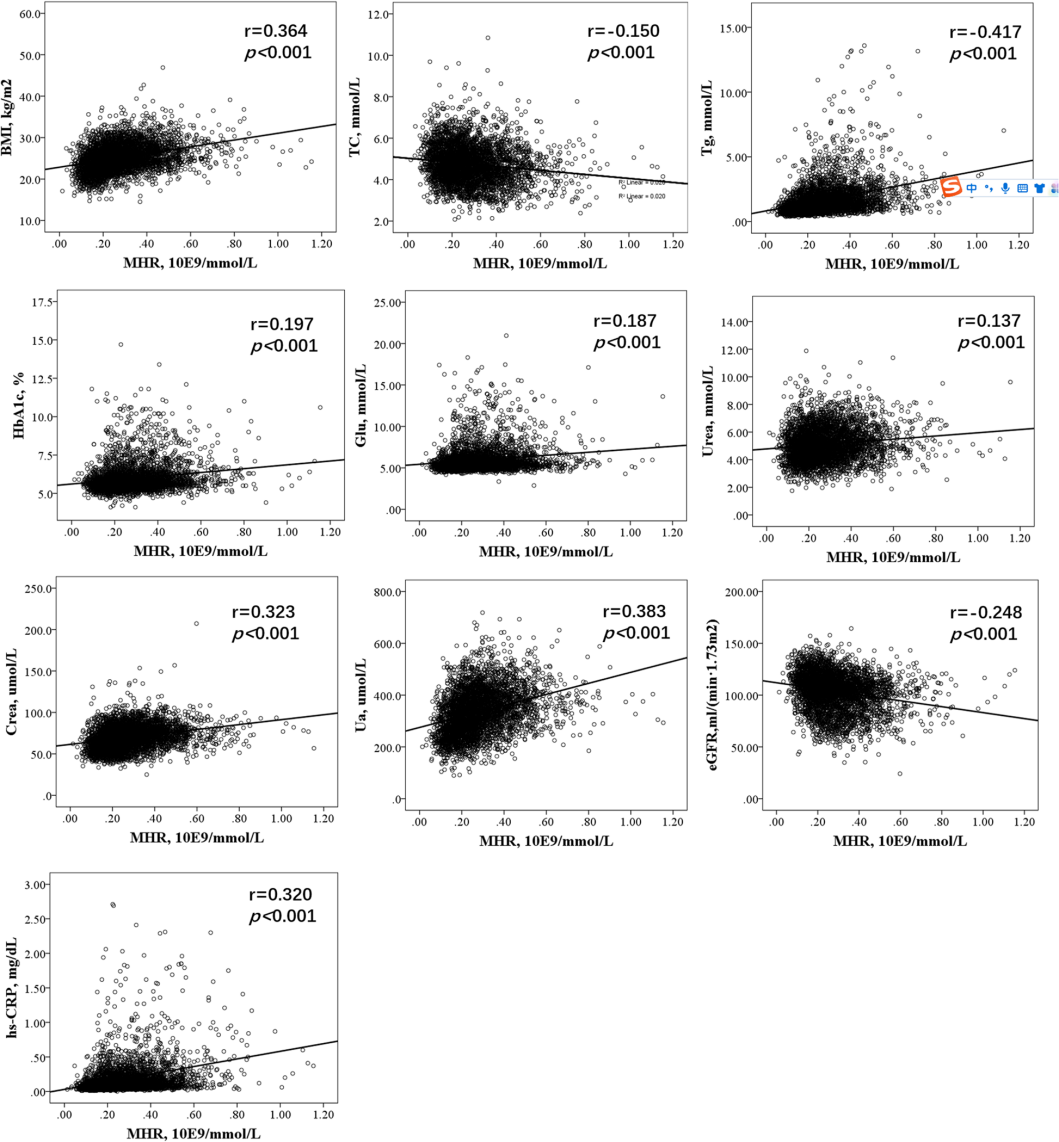
Correlation analysis of MHR and other metabolic indexes. Abbreviations: MHR: monocyte to HDL-C ratio; BMI: body mass index; hs-CRP: high-sensitivity C-reactive protein; Glu: glucose; HbA1c: hemoglobin A1c; LDL-C: low-density lipoprotein cholesterol; TC: total cholesterol; Tg: triglyceride; Crea: creatinine; Ua: uric acid; eGFR: estimated glomerular filtration rate

MHR levels were positively correlated with BMI (*r* = 0.364, *P* < 0.001), hs-CRP (*r* = 0.320, *P* < 0.001), Tg (*r* = 0.417, *P* < 0.001), Crea (*r* = 0.323, *P* < 0.001), eGFR (*r* = − 0.248, *P* < 0.001), Ua (*r* = 0.383, *P* < 0.001) and HbA1c (*r* = 0.197, *P* < 0.001). The levels of TC, Glu, and urea were slightly correlated with the MHR level (*r* = − 0.150, *P* < 0.001; *r* = 0.187, *P* < 0.001; *r* = 0.137, *P* < 0.001, respectively) (as shown in Fig. 1).

### Relationship of MHR levels and severity of carotid plaque in subjects with or without hypertension or diabetes

**Fig. 2:**
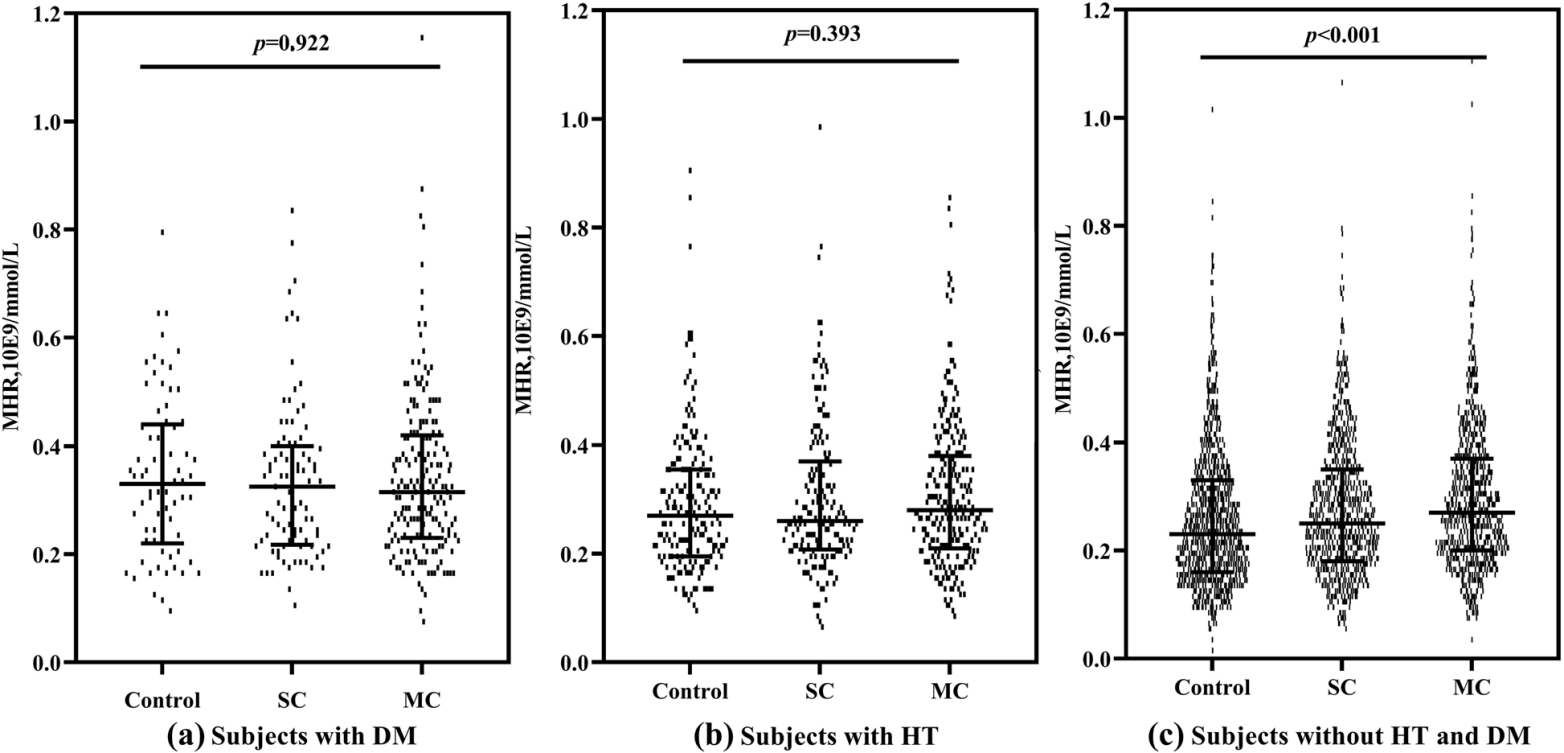
Changes in MHR levels in subjects with or without HT and DM. Abbreviations: HT: hypertension; DM: diabetes

There was no significant difference in MHR levels between subjects with hypertension or diabetes alone (*P* = 0.922 and 0.393, Fig. 2a and b). After the exclusion of subjects with hypertension and diabetes, the MHR level showed differences among the three groups and increased with the multiple occurrences of carotid plaques (*P* < 0.001, Fig. 2c).

As a nonnormal distribution was found in the MHR data, the MHR level in each group is shown as the median (25th percentile, 75th percentile); a nonparametric statistical method (Kruskal-Wallis H test) was used to compare groups.

### Evaluation of the effect of MHR changes on carotid plaque

**Fig. 3:**
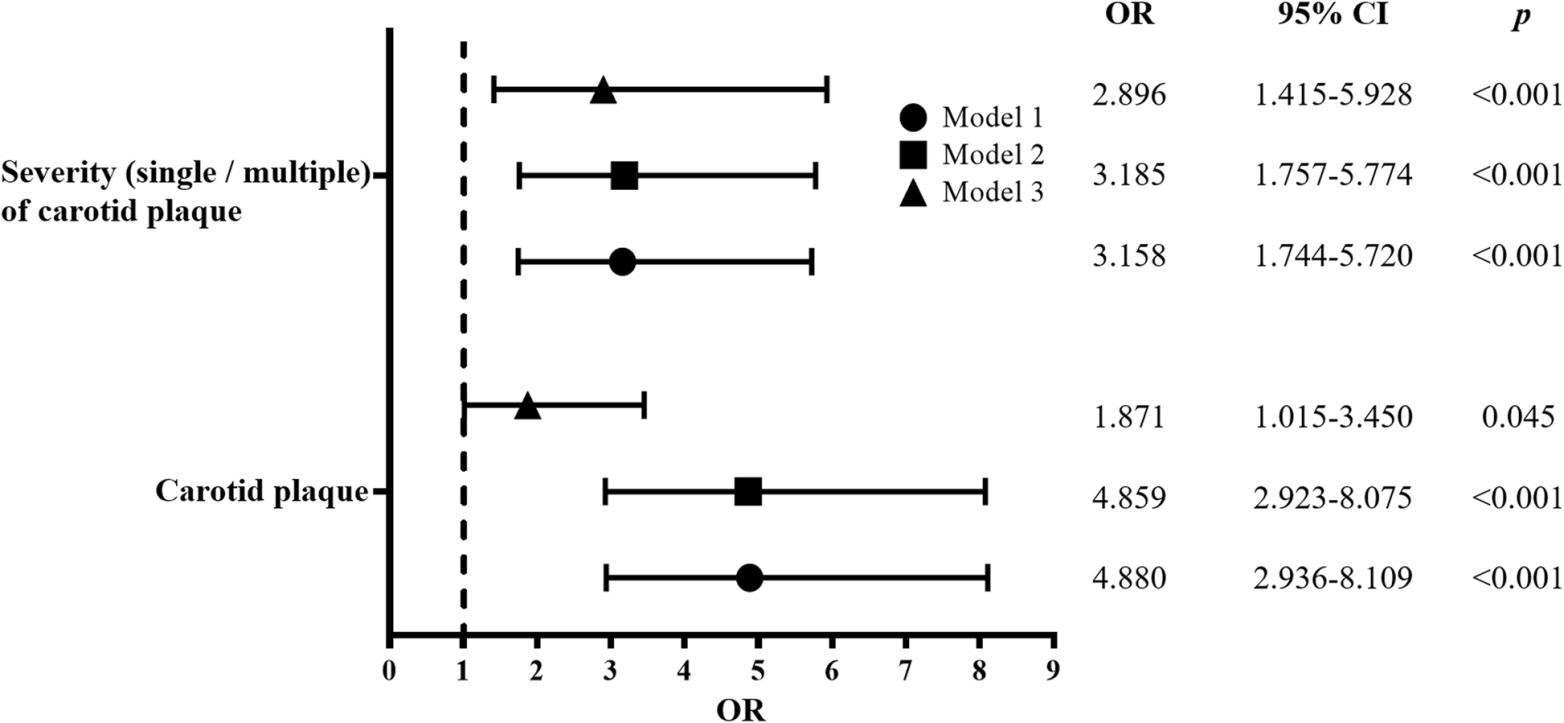
Evaluation of the impact of MHR on carotid plaque by multivariate logistic regression models. Model 1: unadjusted OR; Model 2: adjusted for age and sex; Model 3: adjusted for sex, age, BMI, hypertension, diabetes, urea, Crea, Ua, eGRR, hs-CRP, TC, and Tg. Abbreviations: OR (odds ratio) is shown as the mean (95% confidence interval, CI)

After adjusting for age and sex (in model 2), with the rise in the MHR level, the probability of occurrence of carotid plaque had a 4.859-fold (95% CI: 2.923–8.075, *P* < 0.000) increase; the probability of multiple occurrences of carotid plaques had a 3.185-fold (95% CI: 1.757–5.774, *P* < 0.000) increase. After adjusting for sex, age, BMI, hypertension, diabetes, urea, Crea, Ua, eGRR, hs-CRP, TC, and Tg (in model 3), with the rise in the MHR level, the probability of carotid plaque occurrence had a 1.871-fold (95% CI: 1.015–3.450, *P* = 0.045) increase; the probability of multiple occurrences of carotid plaques had a 2.896-fold (95% CI: 1.415–5.928, *P* < 0.001) increase (Fig. 3).

**Fig. 4:**
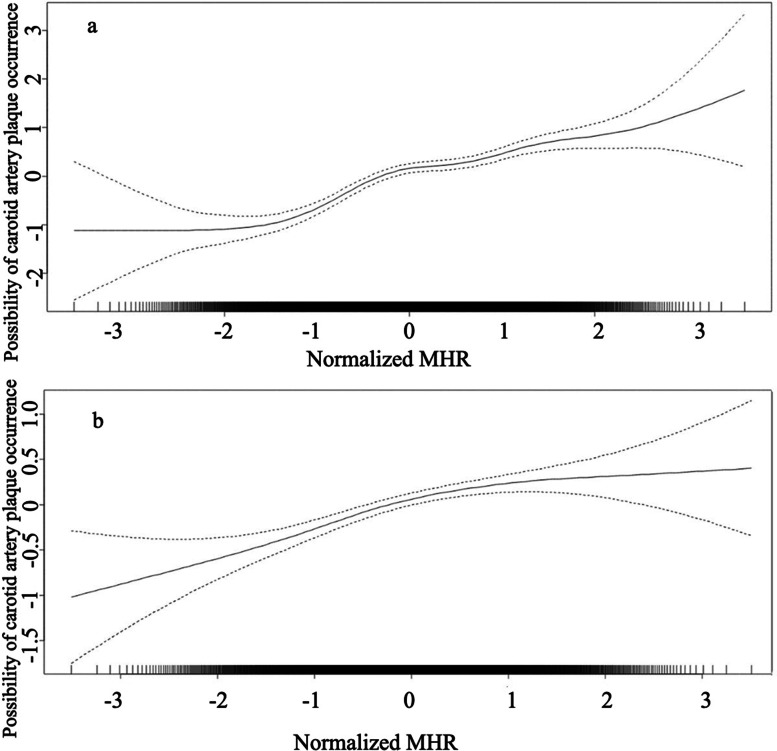
GAM with smooth curve fitting on relationships of MHR levels and likelihood of carotid plaque occurrence. **a** In all subjects; **b** In subjects without hypertension or diabetes. In this figure, due to the nonnormal distribution of MHR, after normalization of MHR, we studied the relationship between the normalized MHR level and the occurrence of carotid plaque; the solid line indicates the mean of estimation of the probability of atherosclerotic plaque occurrence, and the dotted lines serve as pointwise standard errors (SE)

In further analysis, to explore the relationships of MHR levels and the risk of carotid plaque, GAM with a smooth curve fitting was used (Fig. 4). The GAM results indicate that the likelihood of carotid plaque occurrence increased with the increase in normalized MHR; there may be a nonlinear correlation between the increase in MHR level and the occurrence of arterial plaque, which is consistent with the previous regression analysis results.

## Discussion

Our study focused on the association of the MHR with the occurrence and severity of carotid plaque in a physical examination population. Our main findings included the following: first, the results showed that the change in the MHR level was associated with the occurrence of carotid plaque; second, the change in the MHR level was related to the multiple carotid plaques, and the MHR level increased in subjects with multiple carotid plaques. Our results suggest that MHR, as an inflammatory marker, has a suggestive role in the formation and severity of carotid plaques characterized by vascular injury.

Atherosclerosis is considered an inflammatory process and a response to lipid accumulation in the arterial wall [18]. It has been reported that leukocytes pass through endothelial cells and are activated in the intima, which induces the formation of microvessels and makes it easier for plaques to rupture [19]. Kim et al. [20] reported that in asymptomatic people, the increase in monocyte-dominated leukocyte levels increases the risk of cardiovascular disease. The MHR is an indicator of atherosclerosis that evaluates inflammation and dyslipidemia [21]. As reported, the MHR can help to predict ischemic stroke and related complications [22, 23] and is also related to extracranial and intracranial atherosclerotic stenosis [7]. In this study, it was found that there were differences in MHR, lipid, glucose, liver and renal function levels in subjects with different degrees of carotid plaque, suggesting that there were abnormalities in metabolism in people with carotid plaque. Our results found that with the increase in the severity of carotid plaque, the MHR level increased, and other blood indicators also showed differences. Abnormal glucose and lipid metabolism can enhance inflammation, increase the risk of vascular damage, and promote the formation of carotid plaques. The results suggest that the MHR, as an inflammatory indicator, may have an indicative effect on the formation of carotid plaques.

After adjusting for multiple variables (sex, age, BMI, hypertension, diabetes, urea, Crea, Ua, eGRR, hs-CRP, TC, and Tg), in binary logistic regression, with the increase in the MHR level, the probability of occurrence of carotid plaque increased by approximately 2-fold, and in the multiple regression model, the probability of multiple occurrences of carotid plaques increased by approximately 3-fold. As a validation, we used GAM (nonlinear model) to explore whether the effect of MHR on carotid plaque was nonlinear. The results suggested that the incidence of carotid plaque increased with increasing MHR. There is a correlation between the MHR level and the occurrence of arterial plaque, and the correlation may be nonlinear. Consistent with the results of the previous regression analysis, the results of GAM indicated that the MHR level may be positively associated with the occurrence of arterial plaque. This study found that the proportion of subjects with diabetes and hypertension increased with the severity of plaque. This may be caused by disorders of glucose and lipid metabolism in most diabetes or hypertension patients, and the incidence of carotid plaques is increased [24–28]. Thus, in further analysis, we compared subjects with diabetes, hypertension and those without these two diseases. Due to the small number of people suffering from both hypertension and diabetes, no analysis or discussion was made in this study. After the exclusion of subjects with hypertension and diabetes, the MHR level was still associated with the multiple occurrence of carotid plaque, and the MHR level increased in subjects with multiple plaques.

Disorders of lipid and glucose metabolism and the occurrence of diseases have mutual causes and effects. In this study, the MHR was found to be correlated with BMI, Tg, CRP, HbA1c, Crea, eGFR, and Ua. BMI is a commonly used indicator of obesity. Obesity can cause a series of hormones and metabolic disorders, which directly or indirectly lead to abnormal lipid metabolism, diabetes and atherosclerosis. BMI can predict cardiovascular disease at an early stage [29]. BMI also plays an important role in the occurrence of atherosclerosis [30, 31]. CRP can be found in the early stage of atherosclerosis, and CRP levels are closely related to atherosclerotic plaque size and the risk of recurrence after treatment [32]. CRP is also associated with atherosclerotic mediators such as modified LDL [33], activated complements [34] and foam cells [35]. The increase in the serum Tg level is an important reason for the formation of atherosclerotic lesions [36]. With long-term hyperlipidemia, ox-LDL and cholesterol can cause functional damage to the intima of the arteries, and Tg accumulates in endothelial cells and is engulfed by macrophages to form foam cells [37]. The study also found that Ua has proinflammatory effects on vascular smooth muscle cells [38], and elevated Ua levels are considered to be related to abnormal metabolism or organ injury [39]. This result indicates that MHR has a more sensitive effect on the inflammatory state caused by disorders of glucose and lipid metabolism. This study also found that the MHR was correlated with renal function-related indexes (creatinine and EGFR). We speculated that this may be related to the inflammatory state after metabolic disorder, which may cause renal vascular endothelial injury or vascular lesions (such as arteriosclerosis) and then cause changes in renal filtration function.

There are possible mechanistic explanations for MHR changes in carotid plaque development. Monocyte-dominated leukocyte [20] pass through endothelial cells and are activated in the intima, mediating endothelial damage and atherosclerosis [19]. HDL can promote the reverse transport of cholesterol and has vascular protective effects, such as anti-inflammatory, antioxidant, antithrombotic and anti-atherosclerosis effects. It can also inhibit the expression of tissue factor in monocytes by inhibiting p38 activation and inositol phosphate kinase activity [40]. As MHR is a combination of the inflammatory response and lipid metabolism, our results suggest that MHR can be used as a possible marker of plaque formation and severity.

### Study strengths and limitations

The strength of the study is that we well characterized the subjects based on a large population, and corrected for different indexes in models to improve the reliability of the results. There are limitations in our study. The data we collected are from the physical examination population in our hospital. Unfortunately, the medication history of this part of the data is not complete, so we cannot analyze the difference in MHR levels before and after medication to find the relationship between medication and MHR. We hope to study the changes in the MHR before and after treatment in future research to make it more valuable.

## Conclusions

In this study, in clinical and laboratory evaluation, MHR can be used as a potential indicator of inflammatory response and vascular injury. It was found that an increased MHR level may indicate an increased risk of carotid plaque occurrence. Thus, MHR could be used as a possible marker for plaque formation and severity. Identifying patients with more severe plaque from the population with abnormal plaque can prevent further occurrence of more serious diseases.

## Data Availability

The datasets used during and/or analyzed during the current study are available from the corresponding author upon reasonable request.

## Abbreviations

BMI: Body mass index
WBC: White blood cell
M: Monocyte
hs-CRP: High-sensitivity C-reactive protein
Glu: Blood glucose
HbA1c: Hemoglobin A1c
HDL-C: High-density lipoprotein cholesterol
LDL-C: Low-density lipoprotein cholesterol
TC: Total cholesterol
Tg: Triglyceride
Crea: Creatinine
eGFR: Estimated glomerular filtration rate
Ua: Uric acid
