# Correction: The blood monocyte to high density lipoprotein cholesterol ratio (MHR) is a possible marker of carotid artery plaque

**DOI:** 10.1186/s12944-022-01753-4

**Published:** 2022-12-26

**Authors:** Jie Xi, Shasha Men, Jingzhu Nan, Qiuliang Yang, Jin Dong

**Affiliations:** 1 Center of Translational Medicine Research, Medical Innovation Research Department of Chinese PLA General Hospital, Beijing, 100853 China; 2 Department of Clinical Laboratory, the 1st Medical Centre, Chinese PLA General Hospital, Beijing, 100853 China

**Correction: Lipids Health Dis 21, 130 (2022)**

**https://doi.org/10.1186/s12944-022-01741-8**

Following publication of the original article [1], the authors reported an error in Fig. 1: The input method icon was not removed when combining the figures in Fig. 1. Figure 1 should have appeared as shown below.

**Fig. 1:**
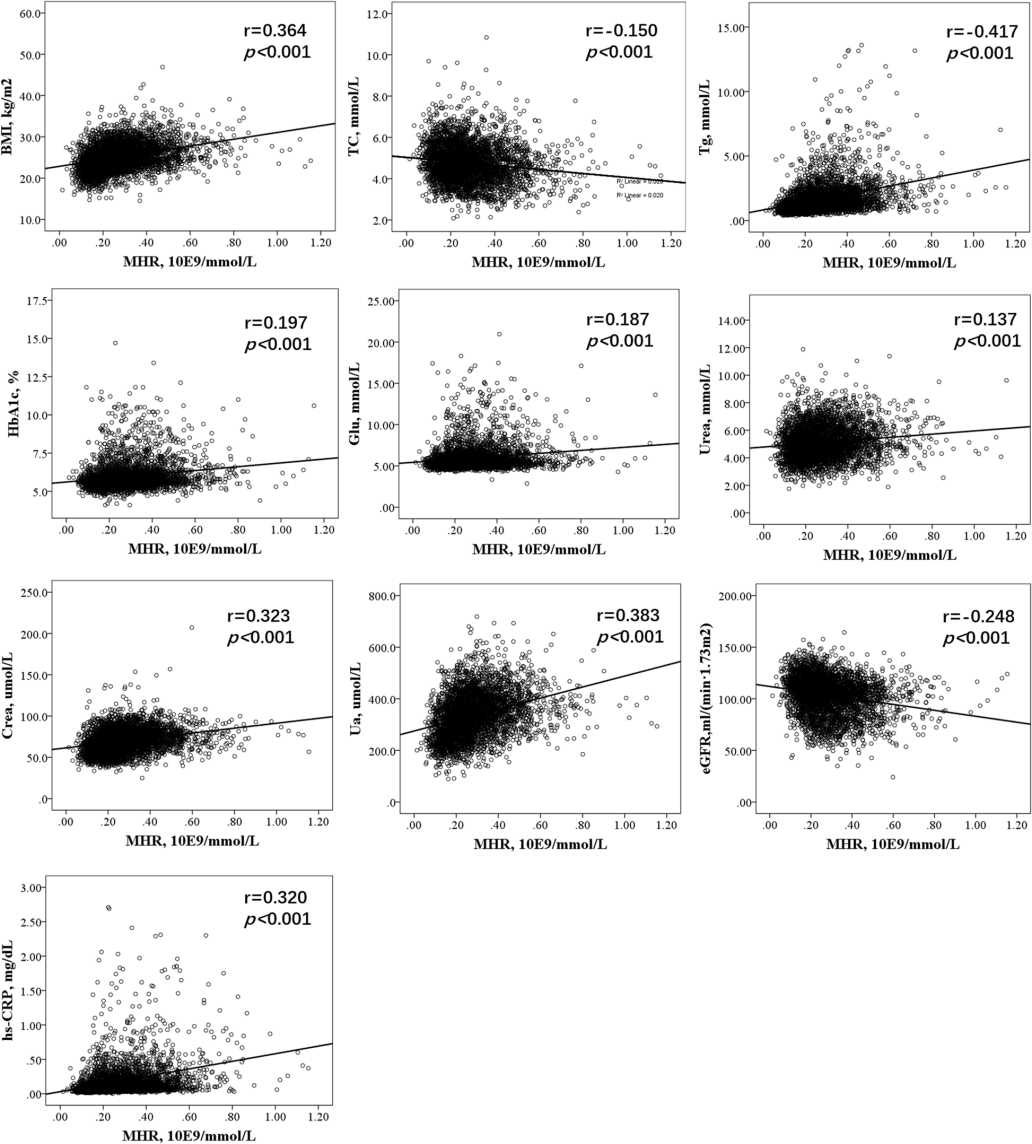
Correlation analysis of MHR and other metabolic indexes. Abbreviations: MHR: monocyte to HDL-C ratio; BMI: body mass index; hs-CRP: high-sensitivity C-reactive protein; Glu: glucose; HbA1c: hemoglobin A1c; LDL-C: low-density lipoprotein cholesterol; TC: total cholesterol; Tg: triglyceride; Crea: creatinine; Ua: uric acid; eGFR: estimated glomerular filtration rate

The original article has been updated.
